# Comprehensive characterization of *Alu*‐mediated breakpoints in germline *VHL* gene deletions and rearrangements in patients from 71 VHL families

**DOI:** 10.1002/humu.24194

**Published:** 2021-03-19

**Authors:** Cathy D. Vocke, Christopher J. Ricketts, Laura S. Schmidt, Mark W. Ball, Lindsay A. Middelton, Berton Zbar, W. Marston Linehan

**Affiliations:** ^1^ Urologic Oncology Branch, Center for Cancer Research, National Cancer Institute National Institutes of Health Bethesda Maryland USA; ^2^ Basic Science Program and Frederick National Laboratory for Cancer Research Frederick Maryland USA; ^3^ Clinical Research Directorate Frederick National Laboratory for Cancer Research Frederick Maryland USA

**Keywords:** *Alu* repeat, genomic rearrangement, germline deletion, hereditary renal cell carcinoma, VHL, von Hippel‐Lindau

## Abstract

Von Hippel‐Lindau (VHL) is a hereditary multisystem disorder caused by germline alterations in the *VHL* gene. VHL patients are at risk for benign as well as malignant lesions in multiple organs including kidney, adrenal, pancreas, the central nervous system, retina, endolymphatic sac of the ear, epididymis, and broad ligament. An estimated 30%–35% of all families with VHL inherit a germline deletion of one, two, or all three exons. In this study, we have extensively characterized germline deletions identified in patients from 71 VHL families managed at the National Cancer Institute, including 59 partial (PD) and 12 complete VHL deletions (CD). Deletions that ranged in size from 1.09 to 355 kb. Fifty‐eight deletions (55 PD and 3 CD) have been mapped to the exact breakpoints. Ninety‐five percent (55 of 58) of mapped deletions involve *Alu* repeats at both breakpoints. Several novel classes of deletions were identified in this cohort, including two cases that have complex rearrangements involving both deletion and inversion, two cases with inserted extra *Alu*‐like sequences, six cases that involve breakpoints in *Alu* repeats situated in opposite orientations, and a “hotspot” PD of Exon 3 observed in 12 families that involves the same pair of *Alu* repeats.

## Graphical abstract

Von Hippel‐Lindau (VHL) is a hereditary multisystem disorder caused by germline alterations in the *VHL* gene. This study characterized 59 partial and 12 complete *VHL* germline deletions in 71 VHL families ranging in size from 1.09 to 355 kb. Exact breakpoints were mapped for 55 partial and 3 complete germline deletions and 95% involved *Alu* repeats at both breakpoints, with a common “hotspot” pair of *Alu* repeats observed in 12 families.

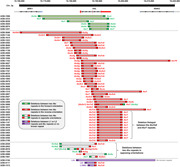

## 1 INTRODUCTION

Patients with von Hippel‐Lindau (VHL) have germline alterations in *VHL* and are at risk for benign and malignant lesions in the kidney, adrenal, pancreas, the central nervous system, retina, endolymphatic sac of the ear, epididymis, and broad ligament (Lonser et al., 2003) Clear cell renal cell carcinoma (ccRCC) arising in VHL patients exhibit loss of function of VHL, usually through loss of the wild‐type allele. *VHL* is also mutated or methylated in up to 90% of ccRCC (Cancer Genome Atlas Research Network, 2013; Gnarra et al., 1994; Nickerson et al., 2008), the most common kidney cancer subtype comprising about 75% of all RCC cases (Linehan & Schmidt, 2019).

Approximately, 30%–35% of VHL patients possess a germline deletion of all or part of *VHL*, rather than a small sequence alteration (Maher & Kaelin, 1997; Richards et al., 1994; Stolle et al., 1998). Germline deletions of 380, 200, and 100 kb in VHL patients were first identified by pulsed‐field gel electrophoresis (Yao et al., 1993) and used to localize the genomic region in which the *VHL* gene was subsequently identified (Latif et al., 1993).

While patients with germline partial deletions (PD), in which one or two exons of *VHL* are deleted, have been reported to be at risk for the development of an aggressive form of renal cell carcinoma (RCC) and mild incidence of pheochromocytoma, patients with complete deletions (CD) are more likely to exhibit mild kidney disease and virtually no incidence of pheochromocytoma (Chen et al., 1995; Franke et al., 2009; Maranchie et al., 2004). Several previous reports have described the sizes, locations, and nature of *VHL* germline deletions (Franke et al., 2009; Maranchie et al., 2004). In 2004, we characterized the deletions of 55 VHL families by fluorescence in situ hybridization (FISH) (Maranchie et al., 2004), and Franke et al. (2009) characterized deletions of 54 families, including 33 that were mapped to the exact nucleotide.

Maranchie et al. (2004) were the first to observe that presence or absence of the adjacent upstream gene, *BRK1* (also known previously as *C3orf10* and *HSPC300*) influences the severity of the RCC phenotype. BRK1 is a subunit of the suppressor of cyclic adenosine monophosphate receptor/Wiskott‐Aldrich syndrome protein family verprolin‐homologous protein actin nucleating complex and is involved in actin and microtubule organization. Depletion of BRK1 by small interfering RNA results in cytoskeleton abnormalities and cytokinesis arrest in cell lines, including clear cell RCC lines (Cascón et al., 2007). In the cohort described by Maranchie et al., the frequency of RCC was 52.3% in *VHL* deletion families that retained *BRK1* versus 18.9% in those that lost *BRK1*. In a cohort of 18 *VHL* deletion probands reported by Cascon et al. (2007), 10 patients who presented with RCC inherited deletions that retained *BRK1* whereas six of eight who did not develop RCC carried deletions that included the *BRK1* gene. In addition, Franke et al. (2009) report RCC in 67% of *VHL* deletion families in which *BRK1* is retained compared with RCC in only 25% of families with *BRK1* deletion.

For over three decades, the Urologic Oncology Branch (UOB) at the National Cancer Institute has followed 402 families with VHL, 112 (28%) of whom have germline deletions. In this study, we have extensively characterized 71 germline *VHL* deletions identified in a cohort of unrelated VHL families managed by the UOB.

## 2 MATERIALS AND METHODS

### 2.1 Patients

Patients were seen at the Urologic Oncology Branch (UOB) of the National Cancer Institute (NCI), National Institutes of Health (NIH) for clinical assessment on institutional review board‐approved protocols and provided written informed consent.

### 2.2 Array‐based comparative genomic hybridization (CGH)

An Agilent custom high‐definition CGH array (Agilent) had been previously designed to assess copy number aberrations in several selected kidney cancer‐associated genes (Benhammou et al., 2011; Vocke et al., 2017). Included within this array were 21 probes selected from the Agilent HD‐CGH database from within the 10.2 kb genomic region containing *VHL* that were computationally preselected to provide an average probe density of ∼2 probes per kb. Within the 50 kb flanking regions 5′ and 3′ to *VHL*, a fade‐out design achieved an average density of ∼1 probe per kb diminishing to an average of ∼1 probe per 40 kb over the entire genome. The custom‐designed arrays were printed on an Agilent 4x44K customer array and processed according to the manufacturer's protocol. Representative patients from 67 VHL germline deletion families were analyzed in this manner; 0.5 μg of patient genomic DNA and 0.5 μg of normal human reference DNA (Promega) were fragmented by AluI/RsaI digestion, labeled with Cy3/Cy5 fluorescent dyes, and hybridized at 65°C for 24 h. Following hybridization and washing, the arrays were scanned using an Agilent Microarray Scanner. Data were extracted with Agilent Feature Extraction Software (v10.7.1.1) and analyzed with Agilent DNA Analytics 4.0 software (v4.0.85). Deletions were calculated as the distance between the first and last probes that lost ~50% of their signal in comparison with the normal signal.

### 2.3 VHL deletion/duplication analysis

All patients from the families with germline deletions identified by the Agilent custom high‐definition CGH array were confirmed by Clinical Laboratory Improvement Amendments (CLIA)‐approved *VHL* deletion/duplication analysis provided by either GeneDx, the Children's Hospital of Philadelphia, or Invitae. Four additional patients were directly evaluated using CLIA‐approved *VHL* deletion/duplication analysis provided by the same companies.

### 2.4 Polymerase chain reaction (PCR) and DNA sequencing

Patient blood DNA was extracted using Promega Maxwell 16 Blood DNA Purification Kits (Promega). Primers were designed adjacent to the estimated deleted region boundaries, and a Qiagen *Taq* PCR Core Kit was used to amplify the deletion boundaries. DNA fragments were gel‐purified using E‐Gel SizeSelect Gels (Life Technologies). DNA sequencing was performed by PCR using a Qiagen Taq PCR Core Kit (Qiagen) according to the manufacturer's specifications, followed by bidirectional sequencing using the Big Dye Terminator v.1.1 Cycle Sequencing Kit (Applied Biosystems) according to the manufacturer's specifications and run on an ABI 3130xl or 3730 Genetic Analyzer (Applied Biosystems). Sanger sequencing was conducted at the CCR Genomics Core at the National Cancer Institute, NIH, Bethesda, MD. Forward and reverse sequences were evaluated using Sequencher 5.0.1 (Genecodes). All deletion breakpoint coordinates and *Alu* locations are based on the GRCh37/hg19 genome build.

## 3 RESULTS

### 3.1 Germline *VHL* gene deletion mapping in 71 families

**Figure 1:**
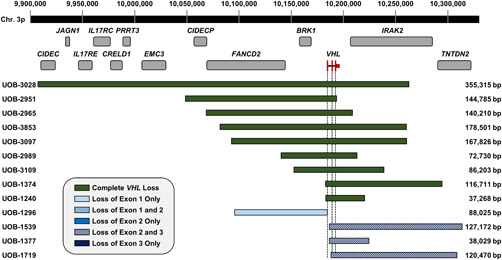
Sizes and ranges of *VHL* germline deletions mapped by CGH. Coordinates are based on the GRCh37/hg19 genome. The extents of deletions were defined by the first and last probe with ~50% loss in the patient compared with normal control. Deletions are grouped based on which exon(s) are deleted. Additional genes that are included in one or more deletions are shown. *VHL* is indicated in red, and the three exons in relation to each deletion are shown by the dotted lines. CGH, comparative genomic hybridization; VHL, Von Hippel‐Lindau

A custom CGH array was used to assess germline copy numbers in representative individuals from 67 unrelated VHL families, who possess germline PD or CD (Benhammou et al., 2011; Vocke et al., 2017). Thirteen of the deletions were mapped by CGH array only; the sizes and ranges are shown in Figure 1 and Table S1. The minimal deletion coordinates are based on the GRCh37/hg19 genome assembly and indicate the first and last CGH probes found to have copy loss for each deletion, and the minimal deletion size is calculated accordingly. Nine of these deletions are CDs and the other four exhibit loss of one or two exons of *VHL*. All 13 of these deletions feature deletion or PD of one or more additional genes upstream or downstream of *VHL*.

**Figure 2:**
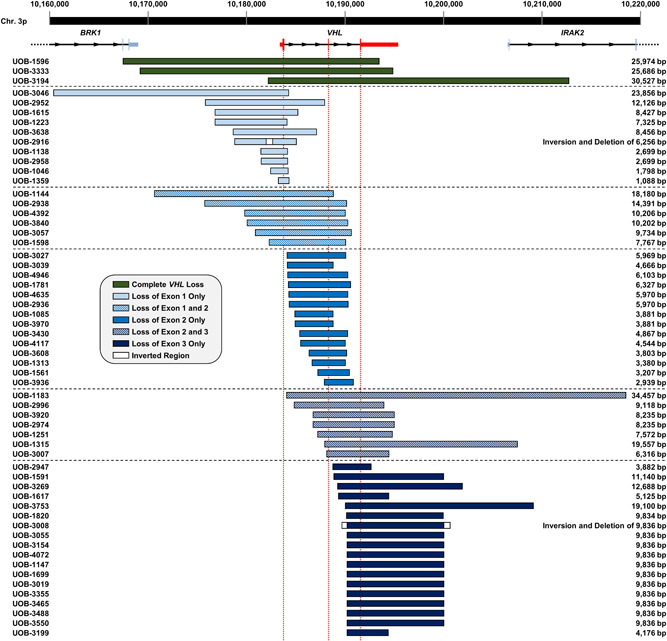
Sizes and ranges of *VHL* germline deletions mapped by CGH and Sanger sequencing. Coordinates are based on the GRCh37/hg19 genome and the sizes are based on the exact deletion breakpoints determined by Sanger sequencing. Deletions are grouped based on which exon(s) are deleted. Deletion/inversion events are depicted with white boxes representing the inverted regions. *VHL* is indicated in red, and the three exons in relation to each deletion are shown by the dotted lines. CGH, comparative genomic hybridization; VHL, Von Hippel‐Lindau

Fifty‐four deletions initially defined by the CGH array were then successfully mapped to the exact nucleotide. Combinations of PCR primers situated in the potentially retained chromosomal regions were used to generate novel amplicons that spanned the deletion and sequenced to identify the deletion breakpoints. An additional four deletions were mapped in a similar manner based on germline results received from a CLIA‐approved *VHL* deletion/duplication genotyping service, as opposed to the CGH array. The chromosomal coordinates of the breakpoints were assigned based on the first divergent nucleotide that was observed on the strand that was sequenced; due to the polyA tails on many *Alu* repeats, the breakpoints often could only be sequenced in one direction. These 58 precisely mapped deletions are shown in Figure 2 and Table S2.

Altogether, 71 *VHL* deletions were mapped, including 12 (16.9%) CDs and 59 (83.1%) PDs, and among these, 58 (3 CDs and 55 PDs) were mapped to the exact breakpoint. The deletions ranged in size from 1088 bp to a minimal deletion of 355,315  bp. The 59 PDs included 11 deletions of Exon 1, 6 of Exons 1 and 2, 14 of Exon 2, 10 of Exons 2 and 3, and 18 of Exon 3. Fifty‐two (73.2%) of these deletions were limited to *VHL*, while the remaining 19 (26.8%) featured deletion of all or part of at least one additional gene. Of note, *BRK1*, upstream of *VHL*, was lost in 10 (14.1%) deletions. Other genes deleted include *FANCD2* (7 deletions), *CICECP* and *EMC3* (2), and *PRRT3*, *CRELD1*, *IL17RC*, *IL17RE*, *JAGN1*, and *CIDEC* (1) upstream of *VHL* and *IRAK2* (15) and *TATDN2* (3) downstream of *VHL*. Families with deletions that extended to include *FANCD2* (and *BRK1*) upstream or *IRAK2* downstream did not demonstrate a phenotype that is noticeably different from those with smaller deletions, other than that deletions involving *FANCD2* have a lower incidence of kidney solids and retinal angiomas, and no pheochromocytomas, similar to what has been seen in the deletion of *BRK1* alone (Franke et al., 2009).

### 3.2 *Alu* repeats define the breakpoints for the majority of germline *VHL* deletions

**Figure 3:**
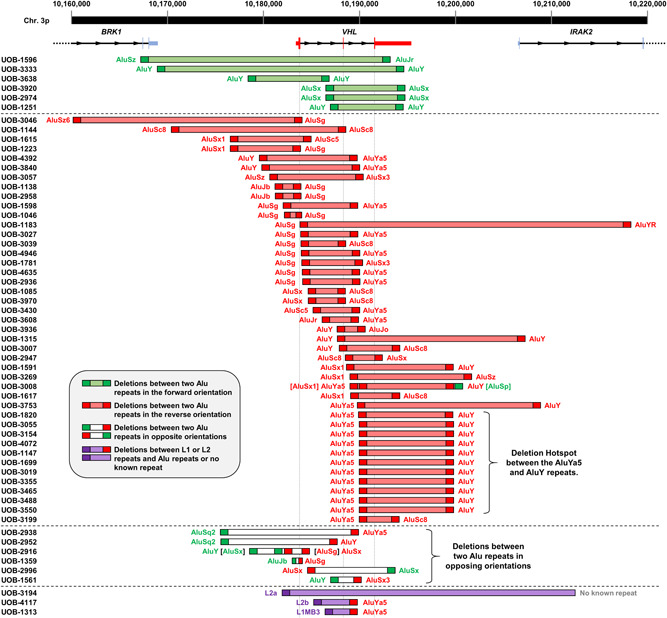
Sizes and ranges of *VHL* germline deletions showing *Alu* repeat involvement. Coordinates are based on the GRCh37/hg19 genome. *Alu* repeats in the forward direction relative to chromosome 3 are shown in green while those in the reverse orientation are shown in red; LINE repeats are shown in purple. Deletions are grouped based on whether the *Alu* repeats are both in the forward direction (light green), both in the reverse direction (light red), in opposite orientations (white), or involving an L1 or L2 LINE repeat (light purple). *VHL* is indicated in red, and the three exons in relation to each deletion are shown by the dotted lines. LINE, long interspersed nuclear element; VHL, Von Hippel‐Lindau

*Alu* repeats are the most common repetitive element in humans; there are about one million copies in the human genome. They are about 300 bp in length and are thus categorized as short interspersed nuclear elements (SINEs) (Hwu et al., 1986). Among the *VHL* deletions that were mapped to the exact nucleotide, 95% (55 of 58) possess *Alu* repeats at both breakpoints and two of the remaining deletions had an *Alu* repeat at one breakpoint. This result is similar to that found by Franke et al. (2009), who observed *Alu* breakpoints in 90% of their deletions. The deletions in relation to their associated *Alu* repeats are depicted in Figure 3.

Of the 55 deletions with *Alu* repeats at both ends, 49 involve *Alu* repeats that are situated in the same orientation on chromosome 3, with six deletions involving *Alu* repeats in the forward orientation (depicted in green in Figure 3) and 43 deletions involving *Alu* repeats in the reverse orientation (depicted in red). Six deletions possess breakpoints in *Alu* repeats situated in opposite orientations (Figure 3).

Three deletions do not have *Alu* repeats at both breakpoints. The deletion identified in proband UOB‐3194 involves an L2a long interspersed nuclear element (LINE) at the left breakpoint and no repeat at the right breakpoint; an L2a repeat and an *AluJr* repeat are 339 and 560 bp, respectively, from the right breakpoint. The deletion present in UOB‐4117 involves an L2b LINE on the left and an *AluYa5* on the right, whereas the deletion in UOB‐1313 involves a LIMB3 LINE on the left and the same *AluYa5* on the right (Figure 3).

Franke et al. (2009) mapped 33 germline VHL deletions by DNA sequencing, and 29 of those deletions involved *Alu* repeats situated in the same orientation while only one involved repeats situated in opposite orientations. LINE or long terminal repeat sequences were responsible for a few of their deletions. Comparison of the deletions found in the Franke et al. (2009) cohort demonstrated some similar deletions: UOB family UOB‐2947 involves the same pair of *Alu* repeats as their Families 26 and 32, UOB‐3638 involves the same pair as their Family 3, UOB‐3430 involves the same pair as their Family 14, and UOB‐4926, UOB‐4635, UOB‐2936, and UOB‐3027 all involve the same pair as their Family 15. However, none of the actual deletion sizes were identical, with the exception that the deletions of UOB‐3027 and Family 15 from *Franke* et al. are both 5969 bp.

The *AluYa5* at chr3:10189995‐10190297 is the most frequently involved repeat element in the UOB cohort; it is involved in 26 breakpoints (44.8%) among our 58 precisely mapped deletions, 14 at the 5ʹ breakpoint and 12 at the 3ʹ breakpoint (Figure 3). Interestingly, Franke et al. (2009) report that this element is also involved in 7 (21.2%) of their 33 deletions. The *AluSg* at 10184023‐10184327 and the *AluY* at 10199822‐10200133 are each involved in 13 breakpoints (22.4%), and the remaining repeats are involved in five or fewer breakpoints.

### 3.3 Combined chromosomal inversion and deletion of VHL involving *Alu* repeats

**Figure 4:**
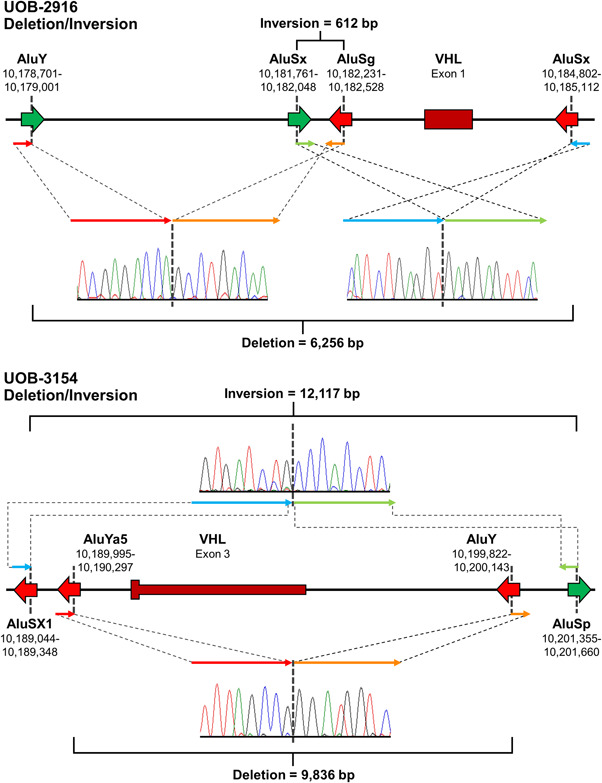
Structures of the deletion/inversion events detected in patients UOB‐2916 and UOB‐3154, respectively, with chromatographs showing the breakpoints. Locations and orientations of the *Alu* repeats are shown. Coordinates are based on the GRCh37/hg19 genome

In sequencing across the breakpoints of families UOB‐2916 and UOB‐3154, additional breakpoints were discovered that were not consistent with simple deletions. Instead, these families possessed chromosomal inversions in addition to the deletions that had been identified by CGH. The structures of these two deletions/inversions are shown in Figure 4. In both cases, the inversion breakpoints and the deletion breakpoints all involved *Alu* repeats. UOB‐2916 had both a deletion and inversion involving *Alu* repeats situated in opposite orientations, while UOB‐3154 had a deletion involving two reverse‐oriented *Alu* repeats and an inversion involving *Alu* repeats situated in opposite orientations (Figure 4). To our knowledge, these are the first deletion/inversion events that have been reported in any VHL families.

### 3.4 A “hotspot” *Alu* repeat‐based deletion of VHL Exon 3

A deletion “hotspot” exhibiting very similar breakpoints involving the same pair of *Alu* repeats, the most frequently involved repeat element *AluYa5* at chr3:10189995‐10190297 and the *AluY* at chr3:10199822‐10200133, was identified in 12 of the Exon 3 deletions, representing 16.9% of the families in our cohort. Interestingly, this *Alu* repeat pairing was not observed at all in the Franke et al. (2009) cohort. Although all *Alu* repeats have an average of 85% homology (Shen et al., 1991), these two particular *Alu* repeats are extremely homologous, having 92.7% identity including 96% identity in the first 175 bp, suggesting that recombination involving this pair may be particularly favorable.

**Figure 5:**
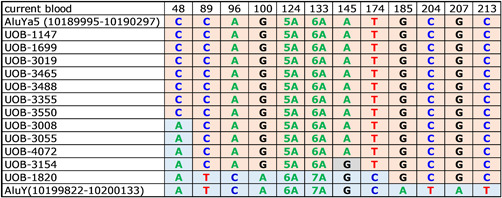
Sequences of the 12 hotspot deletion breakpoints with respect to *AluYa5* 10189995‐10190297 (orange background) and *AluY* 10199822‐10200133 (blue background). UOB‐1147, 1699, 3019, 3465, 3488, 3355, and 3550 form one group with breakpoints that cannot be distinguished, UOB‐3008, 3055, and 4072 form a second group, and UOB‐3154 and UOB‐1820 are unique

To the best of our knowledge, these 12 families are all unrelated. Although it is possible that a founder effect may be responsible for some of the 12 deletions, in at least some cases these deletions appear to be distinct events. Although all 12 deletions involve the same two *Alu* repeats, the actual breakpoints within the *Alu* repeats vary as can be shown by the slight sequence differences in the reconstituted breakpoint region (Figure 5). Seven families (UOB‐1147, 1699, 3019, 3465, 3488, 3355, and 3550) retained the nucleotides, which are unique to the *AluYa5* at all positions (Figure 5). Of these seven families, UOB‐3355 is Caucasian‐European, UOB‐3465 is a Hispanic Central American, and UOB‐1699 is Asian American, while the remaining four families are Caucasian‐American and could potentially be distantly related. Three families (UOB‐3008, 3055, and 4072) retained the A nucleotide of the *AluY* at position 48 while exhibiting the *AluYa5*‐specific sequence at the other positions and are all Caucasian‐American; it cannot be ruled out that these three families could be distantly related. UOB‐3154 also retained the A nucleotide at position 48 and the *AluYa5* sequence at most of the other positions, but features a G at position 145 (Figure 5); this family also possessed the deletion/inversion (Figure 4) that was not seen in the other 11 families and thus represents a unique event. UOB‐1820 retained the *AluY* sequence at positions 48 through 174 and the *AluYa5* sequence at the remaining positions (Figure 5), and thus also represents a unique breakpoint. Chromatographs showing the DNA sequences of this region in all 12 deletions are shown in Figure S1.

Aside from this hotspot deletion, all of the other deletions in this cohort were unique. Four families with a deletion of Exon 2 only (UOB 4946, 4635, 2936, and 3027) had the same pair of *Alu* repeats, but all have different breakpoints and deletion sizes (Table S2). Several pairs of families (UOB‐4392 and UOB‐3840, UOB‐3920 and UOB‐2974, UOB‐3970 and UOB‐1085) shared the same pair of *Alu* repeats, but all had different breakpoints (Table S2). The remaining deletions all involve unique combinations of *Alu* or LINE repeat pairs or were not associated with repeat elements.

### 3.5 Two VHL germline deletions include insertions of *Alu*‐like sequences

Two of the deletions, UOB‐2938, and UOB‐2952, feature extra inserted sequences of 263 and 262 bp, respectively (Figure S2). The extra sequences do not align precisely with any chromosomal region but are highly homologous to the *Alu* repeat family. Interestingly, both sequences are present just 3ʹ to the 5ʹ breakpoint in the *AluSq* at chr3:10175645‐10175927, and both deletions are among the rare deletions observed with the *Alu* breakpoint pair situated in opposite orientations. It is unknown whether these sequences represent insertion of novel *Alu*‐like sequences, possibly via DNA replication errors, or if multiple recombination events have rendered the resulting sequences unable to be aligned directly to the human genome.

## 4 DISCUSSION

Germline deletions of *VHL* represent a significant proportion of all genetic alterations associated with this disorder, and the mapping of these deletions provides important data concerning the spectrum of presentation of VHL and for genetic screening. The predilection for deletions within this region is inherently linked to the significantly increased presence of *Alu* repeats within the region of the *VHL* gene. When the first *VHL* deletion was mapped by Casarin et al. (2006), they demonstrated that both breakpoints occurred within *Alu* repeats. They observed that *Alu* repeats occur approximately once in every 1 kb in the *VHL* region, representing 20%–25% of the *VHL* gene sequence, with the frequency as high as one in every 500–600 bp in some parts of the gene (Casarin et al., 2006). This is much higher than the average *Alu* repeat frequency of one in every 4 kb seen across the genome (Hwu et al., 1986). *Alu*‐mediated recombination has been implicated in many human diseases and it was theorized that *Alu*‐mediated recombination could be a common mechanism for *VHL* gene deletion (Casarin et al., 2006; Deininger & Batzer, 1999). This theory was demonstrated in several additional studies of germline *VHL* deletion (Cascón et al., 2007; Franke et al., 2009; Maranchie et al., 2004). This study confirms that the vast majority of germline deletions are directly associated with the *Alu* repeats within the gene region and identifies a previously unreported hotspot involving two *Alu* repeats with very high sequence homology.

We have previously reported the presence of germline deletions in two other familial forms of kidney cancer, Birt–Hogg–Dubé (BHD), caused by germline mutations in the folliculin (*FLCN*) gene, and hereditary leiomyomatosis and renal cell carcinoma (HLRCC), resulting from germline fumarate hydratase (*FH*) gene mutations (Benhammou et al., 2011; Vocke et al., 2017). Among *FLCN* deletions in four BHD families that were mapped by sequencing, two involved *Alu* repeats in both breakpoints, one had a deletion/inversion event involving *Alu* repeats in two of the four breakpoints, and one had no *Alu* involvement. A germline duplication in *FLCN* likewise did not have *Alu* involvement (Benhammou et al., 2011). Among three HLRCC families with *FH* deletions that were mapped, none had *Alu* involvement (Vocke et al., 2017). In contrast, *Alu* involvement in *VHL* deletion breakpoints is nearly universal, likely due to the unusually high density of *Alu* repeats in the *VHL* genomic region. This may be an explanation as to why such a high proportion (30%–35%) of VHL patients possess a germline deletion, in contrast to a minority of patients in other hereditary kidney cancer syndromes with germline gene deletions (Schmidt et al., 2005; Toro et al., 2003; Wei et al., 2006).

Franke et al. (2009) conducted an analysis of 54 VHL germline deletions, 33 of which were precisely mapped by sequencing. They observed deletions ranging in size from 568 bp to 250 kb, and among the 33 sequenced deletions, 90% involved *Alu* repeats. They found that the single *AluYa5* repeat in the region, which they report as evolutionarily the youngest, was involved in 7 of 33 deletions. In our cohort, this *AluYa5* is involved in an even higher frequency (26/58, 45%) of breakpoints.

Although our results share many similarities with the above study, we observe several novel features. This study identified the first *VHL* deletion hotspot, which was observed in 12 families involving an *AluYa5* and *AluY* pair. Notably, this is the same *AluYa5* element that was involved in 45% of breakpoints in our cohort. This report also provides the first evidence of ~260 bp insertions of *Alu*‐like sequences into deletion breakpoints and identified two novel *VHL* deletion/inversion events, a type of genetic alteration that had not been previously described in VHL. We had previously observed a deletion/inversion event in *FLCN* in a BHD family (Benhammou et al., 2011). The inversions that we observed in both cases were discovered fortuitously; in sequencing PCR products that would represent an expected deletion, additional breakpoints representing additional inversion events were detected. Due to the high frequency of *Alu* repeats in the vicinity of the *VHL* gene and the resultant potential for genomic instability, it is possible that deletion/inversion events are underappreciated and that more such events would be detected by other methods such as sequencing long‐range PCR products or by whole genome sequencing. Furthermore, the possibility exists that an inversion or other complex rearrangement in the absence of a deletion could take place. Such an event would be difficult to detect by conventional CLIA genotyping, which only looks for point mutations or copy number variations. Therefore, for a patient who exhibits clinical manifestations of VHL with no detectable germline alteration, whole‐genome sequencing should be considered to investigate the possibility of an inversion.

In summary, this report describes the largest known cohort to date of *VHL* deletions with extensive characterization. The diverse spectrum of sizes, breakpoints, and *Alu* pair involvement in our study and the studies of others demonstrate the broad range of independent recombination events involving combinations of different *Alu* repeats and other sequences that may contribute to generating germline *VHL* deletions leading to the multisystem phenotype of VHL syndrome.

## CONFLICT OF INTERESTS

The authors declare that there are no conflict of interests.

## WEB RESOURCES

LUMC Mutalyzer: https://mutalyzer.nl/

## Supporting information

Supplementary information.

Supplementary information.

## Data Availability

All identified deletions were submitted to ClinVar (https://www.ncbi.nlm.nih.gov/clinvar/) and are publicly available with continuous accession numbers from VCV000997719 to VCV000997768.
