# Supplementary material for: Comprehensive characterization of Alu‐mediated breakpoints in germline VHL gene deletions and rearrangements in patients from 71 VHL families
Source: Hum Mutat. 2021 Mar 19;42(5):520–9. doi: 10.1002/humu.24194 (PMC8068631; doi:10.1002/humu.24194)
Supplement: Supplementary file 1 — Supplementary information. [file HUMU-42-520-s002.pdf]

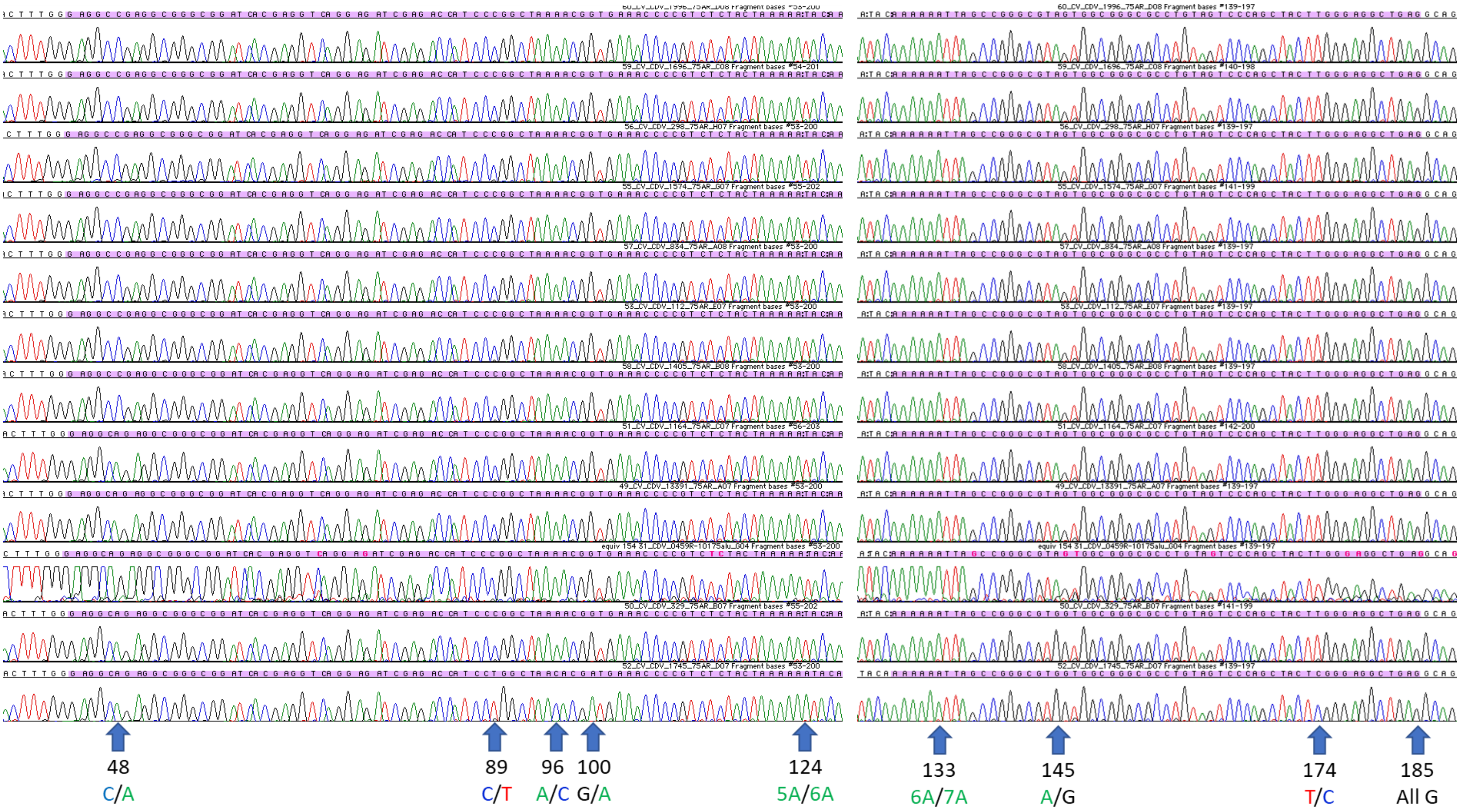

Supplementary Figure S1. Chromatographs showing the DNA sequences of 12 different VHL families that possess a "hotspot" deletion involving the *AluYa5* at chr3:10189995-10190297 and the *AluY* at chr3:10199822-1020013. The positions showing sequence differences between the two *Alu*s are indicated.

UOB-2938 inserted DNA sequence:

TTAGCCAGGCGCTGTGGAAGGCGGTAAGTCCCAGCTACTCGGGAGGCTGA
GGAGGAAGAATCGCTTGAACCCAGGCAGCAGAGGTGGCGGTGCAGTGAGA
TCACAGCCTTCACTTCAGCCTGGGTGATAGAGTGCGACTCTGTCTCAAAA
AAAAAAAAAAATTCCACATATAAGTGAGAACATGCAGTGGTACTTCTTTT
TTTCTTTTTCTTTCTTTTTTTTTTGAGACGGAGTTTCACTCTTGTTGCCA
AGAGGAGTGCAGC

UOB-2952 inserted DNA sequence:

TTGAACCCAGGAGGCGGAGGTTGCAGTGAGCTGAGATCATGTCACTGCAC
TCCTCTTGGGCAACAAGAGTGAAACTCCGTCTCAAAAAAAAAAAAAGAAA
GAAAAAGAAAAAAAGAAGATAAATACCKGCWTKTTYKCRSTTATATGTGG
AATTTTTTTTTTTTTTTTTTGAGACAGAGTCTCACTCTATCACCCAGGCT
GAAGTGCAGTGGCGTGATCTCAGCTCACCGCCACCTCTGCTGCCTGGGTT
CAAGCGATTCTT

Supplementary Figure S2: The unique Alu-like sequences that have been inserted at the breakpoints of the deletions in UOB-2938 and UOB-2952, respectively.

Family ID	Deletion type	Additional deleted/partially deleted genes	Hg19 minimal deletion	HGVS annotation	Minimal deletion size
UOB-3028	CD	CIDEC, JAGNI, IL17RE, CRELD1, PRRT3, PRRT3AS1, EMC3, EMC3-AS1, CICECP, FANCD2, FANCD2OS, BRK1, IRAK2	chr3:9907355-10262669	NC_000003.11:g.(?_9907355)_(10262669_?)del	355,315 bp
UOB-2951	CD	EMC3, EMC3-AS1, CIDECP, FANCD2, FANCD2OS, BRK1	chr3:10048652-10193436	NC_000003.11:g.(?_10048652)_(10193436_?)del	144,785 bp
UOB-2965	CD	FANCD2, FANCD2OS, BRK1, IRAK2	chr3:10068504-10208713	NC_000003.11:g.(?_10068504)_(10208713_?)del	140,210 bp
UOB-3853	CD	FANCD2, FANCD2OS, BRK1, IRAK2	chr3:10081500-10260000	NC_000003.11:g.(?_10081500)_(10260000_?)del	178,501 bp
UOB-3097	CD	FANCD2, FANCD2OS, BRK1, IRAK2	chr3:10092365-10260190	NC_000003.11:g.(?_10092365)_(10260190_?)del	167,826 bp
UOB-1296	PD	FANCD2, FANCD2OS, BRK1	chr3:10095290-10183314	NC_000003.11:g.(?_10095290)_(10183314_?)del	88,025 bp
UOB-2989	CD	FANCD2, FANCD2OS, BRK1, IRAK2	chr3:10140054-10212783	NC_000003.11:g.(?_10140054)_(10212783_?)del	72,730 bp
UOB-3109	CD	BRK1, IRAK2	chr3:10152535-10238737	NC_000003.11:g.(?_10152535)_(10238737_?)del	86,203 bp
UOB-1374	CD	IRAK2, TATDN2	chr3:10182693-10299403	NC_000003.11:g.(?_10182693)_(10299403_?)del	116,711 bp
UOB-1240	CD	IRAK2	chr3:10182963-10220230	NC_000003.11:g.(?_10182963)_(10220230_?)del	37,268 bp
UOB-1539	PD	IRAK2, TATDN2	chr3:10184329-10311500	NC_000003.11:g.(?_10184329)_(10311500_?)del	127,172 bp
UOB-1377	PD	IRAK2	chr3:10184329-10222357	NC_000003.11:g.(?_10184329)_(10222357_?)del	38,029 bp
UOB-1719	PD	IRAK2, TATDN2	chr3:10187494-10307963	NC_000003.11:g.(?_10187494)_(10307963_?)del	120,470 bp

Supplementary Table 1: aCGH-mapped *VHL* deletion sizes and locations

PD – Partial Deletion, CD – Complete Deletion
